# Pedicle screw loosening: the value of radiological imagings and the identification of risk factors assessed by extraction torque during screw removal surgery

**DOI:** 10.1186/s13018-018-1046-0

**Published:** 2019-01-07

**Authors:** Xiaoliang Wu, Jiawei Shi, Jinyan Wu, Yongquan Cheng, Kaiwen Peng, Jianting Chen, Hui Jiang

**Affiliations:** 1 Department of Spinal Surgery, Nanfang Hospital, Southern Medical University, Guangzhou, China; 2 Respiratory Department, Nanfang Hospital, Southern Medical University, Guangzhou, China

**Keywords:** Pedicle screw fixation, Screw loosening, Extraction torque

## Abstract

**Background context:**

Pedicle screw loosening is a common complication after spine surgeries. Traditionally, it was assessed by radiological approaches, both X-ray and CT (computed tomography) scan, while reports using mechanical method to study screw loosening after spine surgery are rare. The primary objective was to study the prevalent of pedicle screw loosening according to extraction torque during screw removal surgery and access the sensitivity and specificity of both X-ray and CT scan for diagnosing screw loosening. The second objective was to identify the risk factors for low extraction torque of pedicle screw that might lead to loosening.

**Methods:**

Thirty-three patients who underwent pedicle screw removal surgery after at least 2 years from primary surgery were evaluated preoperatively for fixation stability by X-ray and CT scan. In total, 236 screws were taken out, and the extraction torque data was recorded and analyzed to identify the sensitivity and specificity of both imaging studies for screw loosening. Furthermore, risk factors that might contribute to low extraction torque were also studied.

**Results:**

The mean extraction torque of removed screws was 1.55 ± 1.00 Nm; a torque force of less than 1.02 Nm was used to define a screw as loosened. According to such criterion, the loosening rate was found to be 33%. X-ray had a sensitivity of 24% and a specificity of 98%, while CT scan had a sensitivity of 22% and a specificity of 96%. Extraction torque of pedicle screws inserted in fractured vertebrae was significantly lower than those in non-fractured vertebrae (*p* = 0.009); meanwhile, screws of non-fusion surgery had lower extraction torque when compared to those in fusion surgery (*p* = 0.001). BMD (bone mineral density) and age had low but significant linear relationship with screw extraction torque (*p* = 0.01, *R*^2^ = 0.304; *p* = 0.045, *R*^2^ = 0.123).

**Conclusions:**

Our findings showed that both X-ray and CT scan had high specificity for screw loosening detection, but their sensitivities were relatively low. Surgeons needed to be more cautious when assessing screw loosening merely according to radiological examination, and aware of that screws in fractured vertebrae or non-fusion surgery were vulnerable to loosening.

## Background

Pedicle screw fixation is widely used in spine surgery for numbers of indications, such as degenerative disease, trauma, tumor, infection, and deformity. It reduces the range of motion of the stabilized spine, increases the fusion rate, and is generally considered to be safe with relatively low complication rate associated with the device [1, 2].

One of the typical complications widely reported in literatures is screw loosening, which may lead to fixation failure and require revision surgery [3]. The key factor regarding investigation of screw loosening is the assessment of whether a screw is loosened or not, which is traditionally based on radiological approaches [3]. The diagnostic criteria for loosening developed by X-ray include the radiolucent area (thicker than 1 mm) around screw [4–9] and the “double halo” [4, 10] defined as the presence of radiolucent area and radiopaque rim at the same X-ray. Nevertheless, the specific details regarding X-ray criteria of loosening were not described in most papers, suggesting that the subjective viewpoints of surgeon and radiologist played an important role. Furthermore, the sensitivity and specificity of the X-ray criteria of loosening could be confounded by many factors, such as metal image artifact, intestinal gas, and display angle. CT scan was also employed by some studies and considered the diagnostic imaging modality of choice for detection of screw loosening [4, 9, 11, 12]. Ohtori et al. used both CT scan and X-ray to assess screw loosening, and their results showed that CT scan was significantly more sensitive than X-ray [11]. Nevertheless, like X-ray, the details about how the screw loosening was evaluated by CT scan were obscure and the assessment was subjective too. In general, both X-ray and CT scan lack uniform and explicit standard.

The limitation of radiological approaches led to a great variety of screw loosening rate in literatures. Some papers showed relatively low loosening rate, less than 1% in non-osteoporotic patients evaluated by X-ray [2, 5, 13], while other studies indicated a much higher rate of loosening [8, 12, 14, 15]. Roellinghoff reported that in 64 patients treated with multilevel pedicle screw fixation, 35(54.69%) patients showed radiographic signs of screw loosening [16]. The loosening rate was expected to be even higher in osteoporotic patients [17, 18]. Therefore, the actual circumstances of pedicle screw loosening are not acknowledged due to the various diagnostic criteria and conflicting reports.

Extraction torque, as an objective mechanical indicator, has been used to evaluate the mechanical fixation of pedicle screws in animal model [19]. However, this indicator has seldom been used to study pedicle screw loosening after spine surgeries, especially regarding its relationship with radiological findings. Sanden and colleagues [20], in a cohort study of 21 patients who underwent pedicle screw removal surgery, reported that the radiolucent zones around pedicle screws was associated with lower extraction torque, but they did not employ CT scan or evaluate the risk factors for loosening according extraction torque. Several factors, such as osteoporosis or osteopenia, non-fusion surgery, and long segment fixation, were considered to be related with screws loosening based on imaging study [3]. To the best of our knowledge, no study has evaluated such risk factors according to the extraction torque in vivo. Therefore, we intended to investigate the pedicle screw loosening rate using extraction torques during instrumentation removal surgery, and compare it with X-ray and CT image findings. Meanwhile, by using extraction torque data, we analyzed the risk factors of screw loosening.

## Patients and methods

This was a prospective designed single-center study. Patients who underwent pedicle screw removal surgery were screened for eligibility. The indications for screw removal included the following: (1) pedicle screw fixation for thoracolumbar fracture without fusion and imaging confirmed solid fracture union, (2) patients required screw removal that presented persistent axial para-midline back pain to palpation or abnormal foreign body sensation due to pedicle fixation with imaging confirmed solid fusion, and no other cause found, e.g., infection. Those with significant pedicle malplacement or destructive spine disorders, such as bone metabolic disease, were excluded from our study. Informed consent was obtained from all individual participants included in the study.

**Fig. 1:**
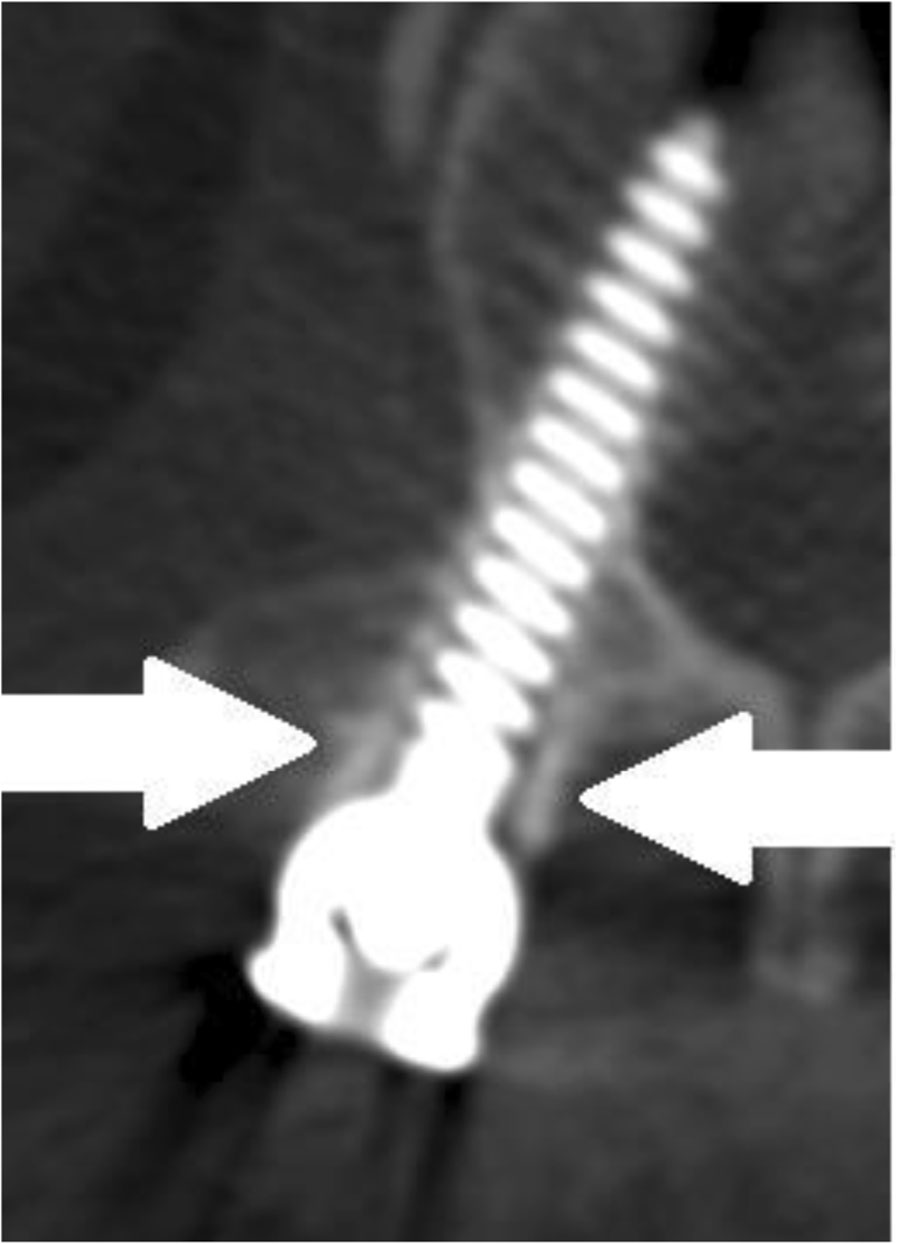
Typical CT image of metal artifact around screw tail. This picture shows metal artifact around screw tail in CT scan, which may confuse the interpretation of screw loosening

The titanium alloy pedicle screws (Johnson&Johnson, USA; Medtronic, USA; Stryker, USA; Kanghui Med, China; FULE, China) with diameter of 4.0 to 6.5 mm and length of 30 to 55 mm were used in the primary surgeries. Conventional lateral and anteroposterior radiographs were taken before and at 3 to 6 months after primary surgery. The same X-ray and CT scan were scheduled before screw removal surgery to evaluate fracture union and spine fusion, as well as stability of instrumentation. Bone mineral density (BMD) was tested using dual energy X-ray absorptiometry. The maximum extraction torque was recorded while unscrewing the screw using torque gauge with a range of 0.06 to 6.00 Nm (Park Tool, China). The same surgeon (J.S. senior resident) with sufficient practice made all the extraction torque recordings. The radiographs were evaluated by a senior spinal surgeon (X.W. senior attending doctor), who was blinded to the extraction torque and patient information. X-ray criteria for screw loosening were a radiolucent zone surrounding the screw thicker than 1 mm and/or the “double halo” sign. CT scan criterion of loosening was a no signal zone surrounding the whole body of screw on the CT image. Because of metal artifact, no signal zone was usually seen around screw tail, which could not be interpreted as screw loosening (Fig. 1).

All values are given as the mean ± SD. Statistical analyses were performed using the IBM SPSS Statistics 13.0 (Chicago, USA). The Chi-square test was used for categorical variables. Mann-Whitney *U* tests or Kruskal-Wallis test was used for continuous variables. TwoStep Cluster was used to clustered screws by torque. We constructed a linear regression model with Pearson correlations analysis to assess whether clinical data, such as age and BMD, were correlated with the extraction torque of screw. The level of statistical significance was selected to be *p* = 0.05.

## Results

**Fig. 2:**
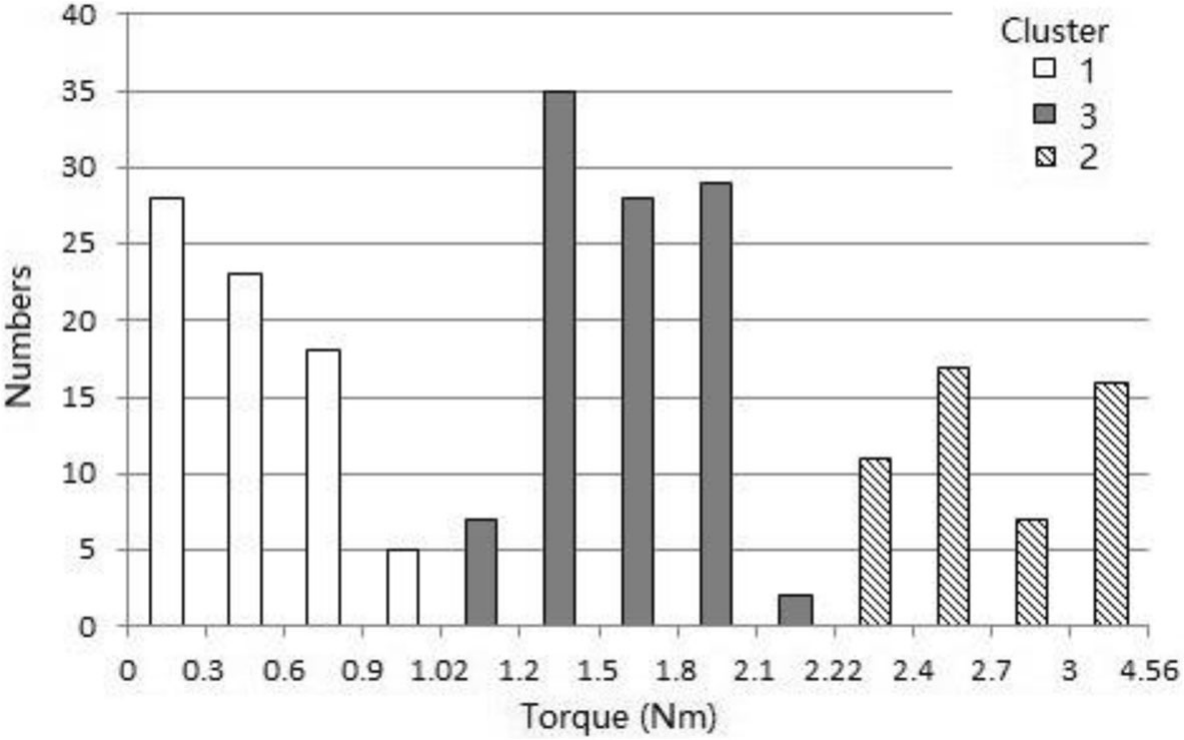
The distribution of screws in three clusters based on extraction torque by Twostep Cluster. By using Twostep Cluster, the distribution of extraction torque of pedicle screws showed two prominent peaks and one valley

Thirty-three patients, 10 females, and 23 males, aged from 17 to 66 years (average age 38 years) at the time of implant removal, were included; patient characteristics are shown in Table 1. The minimal time interval between primary surgery to screw removal was 18 months. Two hundred thirty-six pedicle screws were extracted in total, including 86 in thoracic spine, 138 lumbar, and 12 sacral. One hundred thirty of them were polyaxial and 106 were monoaxial. Four breaking screws were detected. Details of the fixation segments are shown in Table 2. Due to inadequate exposure of screw and malfunction of screw tail, 6 screws had no reading of extraction torque. Therefore, the reading of extraction torque was taken in 226 screws. The mean torque of screws was 1.55 ± 1.00 Nm. The data distribution showed two prominent peaks and one valley. There is a dramatic decline in screw numbers between 0.90 and 1.20 Nm. The screws were clustered into three clusters based on torque by Twostep Cluster as shown in Fig. 2. The cutoff values were 1.02 and 2.22 Nm. Given such result, the torques of 36 pedicle screws in 8 patients were measured during screw insertion as normal match group. The mean torque of the newly implanted screws was 2.73 ± 0.75 Nm (95% confidence interval 1.23 to 4.22). Since 1.02 Nm was significantly less than the 95% confidence interval of newly implanted screws, the pedicle screw loosening based on torque was defined as screws with an extraction torque less than or equal to 1.02 Nm. According to this criterion, there were 74 (33%) loosening screws.

**Table 1:** Patients’ characteristics

Patient characteristics	Overall (*n* = 33)
Age (years)	38.2 (SD 13.8) (17 to 66)
Gender	
Females	10 (28%)
Males	23 (72%)
Height (cm)	163.6 (SD 7.2)
Weight (kg)	61.9 (SD 14.3)
BMI (kg/m^2^)	22.9 (SD 4.0)
Diagnosis	
Facture	16
Degenerative disc diseases	9
Scoliosis	8
Number of patient with X-ray	33
Number of patient with CT scan	28
BMD (g/cm^2^)	0.98 (SD 0.13)
Osteoporosis	1
Persisting pain/foreign body sensation	18
Recovery after removal surgery	16
Time to implant removal (months)	36.4 (18 to 77)
Surgical duration (min)	125 (SD 90)
Fusion in primary surgery	19
Hospital stay (days)	8.4 (SD 3.3)

*SD* standard deviation, *BMI* body mass index, *BMD* bone mineral density

**Table 2:** Detail of removed pedicle screws

Screw characteristics	Overall (*n* = 236)
Valid torque data	226 (95.8%)
Screw breakage	4 (1.7%)
Undetectable	6 (2.5%)
Thoracic spine	86 (36.4%)
t2-t9	38 (16.1%)
t10	6 (2.5%)
t11	10 (4.2%)
t12	32 (13.6%)
Lumbar spine	138 (58.5%)
l1	33 (14.0%)
l2	38 (16.1%)
l3	19 (8.1%)
l4	24 (10.2%)
l5	24 (10.2%)
Sacral(S1)	12 (5.1%)
Polyaxial screw	130 (55.1%)
Monoaxial screw	106 (44.9%)
Short segment	36 (9 patients)
Multiple segments(≥ 3)	200 (24 patients)

Using X-ray radiographs taken the day before removal operations, radiolucent area (wider than 1 mm) around the screw and/or the double halo were detected in 20 screws. Using CT scan, the no density zones were found surrounding 17 screws. As shown in Tables 3 and 4, the X-ray criteria of loosening had a sensitivity of 24% and a specificity of 98%, while the CT scan criterion of loosening had a sensitivity of 22% and a specificity of 96%. There was no significant difference in sensitivity (*p* = 0.863) or specificity (*p* = 1.00) between X-ray and CT scan. The mean torque of screws, which were diagnosed as loosening by X-ray, was 0.53 ± 0.65 Nm, which was significantly lower *(p* < 0.0001) than others (1.65 ± 0.98). While the mean torque of loosened screws diagnosed by CT was 1.03 ± 1.00 Nm, which was also significantly lower *(p* = 0.008) than others (1.71 ± 1.00 Nm).

**Table 3:** Fourfold data of X-ray criteria indicated X-ray had a sensitivity of 24% and a specificity of 98%

X-ray	Loose	Not loose	*p* value
Positive	17 (24%)	3 (2%)	< 0.001^a^
Negative	53 (76%)	153 (98%)	

Positive—radiolucent area (thicker than 1 mm) and/or the double halo around the screw

^a^Chi-square test

**Table 4:** Fourfold data of CT criteria indicated CT scan had a sensitivity of 22% and a specificity of 96%

CT scanning	Loose	Not loose	*p* value
Positive	12 (22%)	5 (4%)	0.001^a^
Negative	43 (78%)	107 (96%)	

Positive—no density zones around screw

^a^Chi-square test

**Fig. 3:**
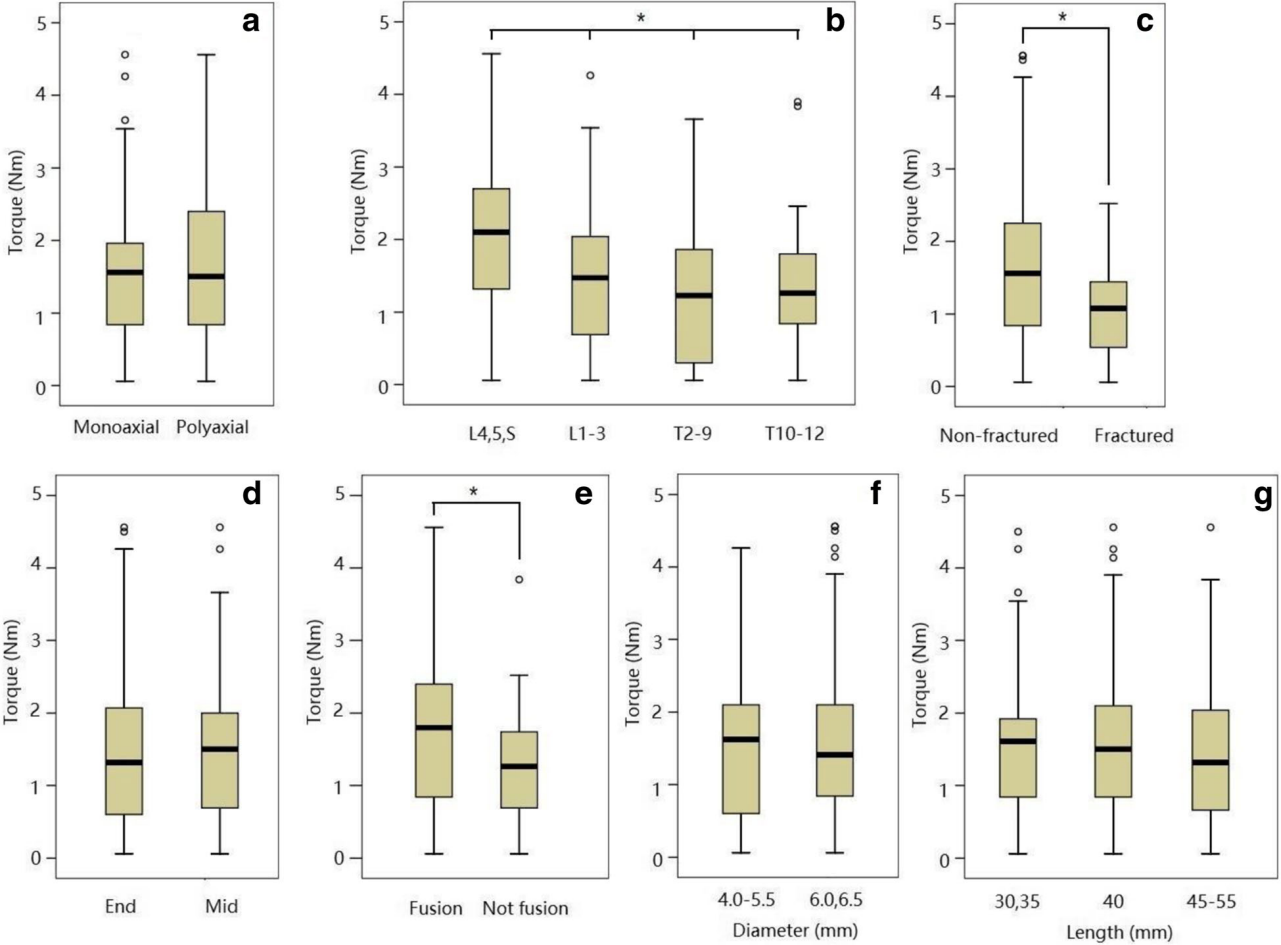
Analyzing risk factors for low extraction torque of pedicle screw. **a** No significant difference was found between polyaxial and monoaxial screws. *p* = 0.673, Mann-Whitney Test. **b** There were significant differences of screw extraction torque among different fixation segments. Screws inserted in the lumbosacral junction (L4, L5, and S1) showed the highest torque. *L* lumbar vertebrae, *S* sacrum, *T* thoracic vertebrae. *p < 0.001, Kruskal-Wallis test. **c** Screw placed in fractured vertebrae showed significantly lower extraction torque than those in non-fractured vertebrae. *p = 0.009, Mann-Whitney Test. **d** There was no significant difference regarding extraction torque between screws placed in the end segments and those in the mid segments. *p* = 0.437, Mann-Whitney Test. **e** Extraction torques of pedicle screw after fusion surgery (*n* = 142) were significantly higher than those in non-fusion surgery. *p = 0.001, Mann-Whitney Test. **f** No significant difference of extraction torque was found among different screw diameter. *p* = 0.988, Mann-Whitney Test. **g** No significant difference of extraction torque was found among different screw length. *p* = 0.746, Kruskal-Wallis test

The risk factors of screw loosening were analyzed. Regarding extraction torque, there was no significant difference between polyaxial and monoaxial screws (*p* = 0.673) as shown in Fig. 3a. Significant difference was found between different segments (*p* < 0.001) as shown in Fig. 3b. The mean torque of screws placed in the lumbosacral junction (L4, L5, and S1, *n* = 54) was 2.14 ± 1.12 Nm, which was significantly higher than those placed in other segments. There were 26 screws placed in 13 fractured vertebrae. The mean torque of screws placed in fractured vertebrae was 1.03 ± 0.63 Nm, which was significantly lower (*p* = 0.009) than those in non-fractured vertebrae, as shown in Fig. 3c. The screws at the ends of implant were supposed to share more strain and be vulnerable to loosening [3]. However, in 24 patients who underwent multilevel (more than two segments) instrumentation, there was no significant difference regarding extraction torque (*p* = 0.437) between screws placed in the end segments (*n* = 88) and those in the mid segments (*n* = 103), as shown in Fig. 3d. Patients with fusion surgery were considered to get better stability than those without fusion; hence, screws in fused spine might share lower strain and have higher torque. As expected, extraction torques of pedicle screw after fusion surgery (*n* = 142) were significantly higher than those in non-fusion surgery (*n* = 84) (*p* = 0.001), as shown in Fig. 3e. There was no significant difference regarding the diameter or length of screws as shown in Fig. 3f, g.

**Fig. 4:**
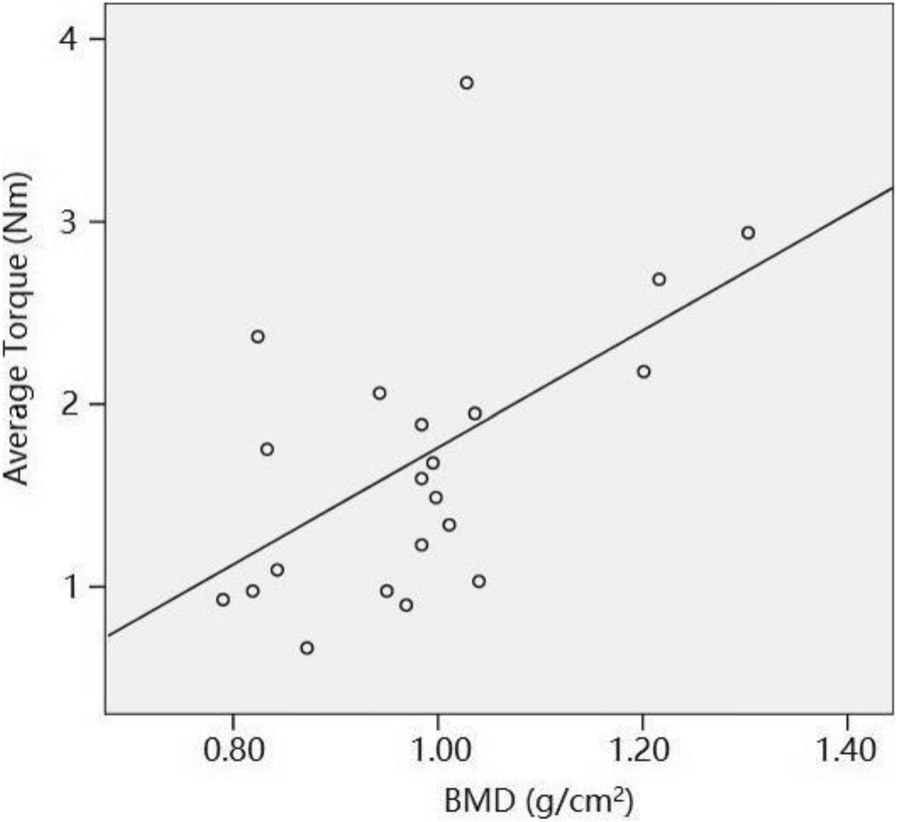
Scatter diagram of patients’ average torques and BMD. The results showed a low but significant linear correlation between extraction torque and BMD. *p* = 0.01, *R*^2^ = 0.267, *F* = 8.296, Linear regression analysis

**Fig. 5:**
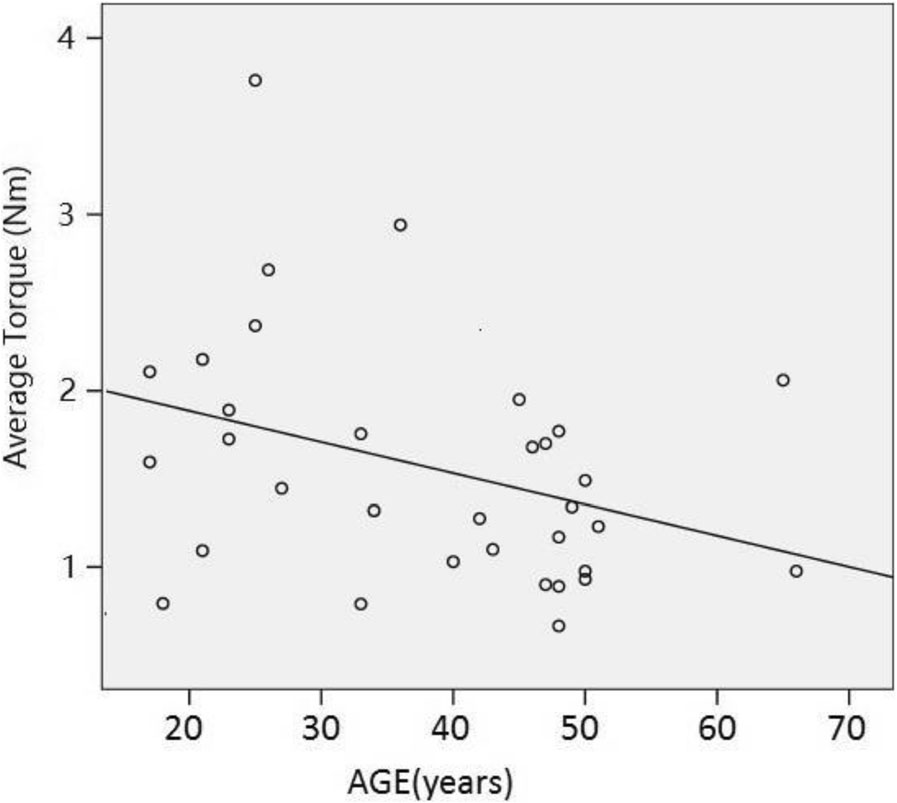
Scatter diagram of patients’ average torques and ages. The results showed a low but significant linear correlation between extraction torque and patients’ age *p* = 0.045, *R*^2^ = 0.123, *F* = 4.345, Linear regression analysis

Linear regression analysis was used to estimate the relevance between the extraction torque of pedicle screw and other clinical findings. Our results showed a low but significant linear correlation between extraction torque and BMD (*p* = 0.010, *R*^*2*^ = 0.304, *F* = 8.296) as well as age (*p* = 0.045, *R*^*2*^ = 0.123, *F* = 4.345), indicating pedicle screw in aged patients or those with low bone density may be less stable according to mechanical measurement, as shown in Figs. 4 and 5. There was no linear correlation between extraction torque and patients’ height (*p* = 0.848), weight (*p* = 0.196), BMI (*p* = 0.125), and time interval between the primary surgery and screw removal surgery (*p* = 0.965).

## Discussion

Pedicle screw loosening has been widely reported as one of the concerning complications after spine instrumentation surgery, which may require revision surgery [21]. But the reported data regarding screw loosening were traditionally based on radiological observation, which could be subjective and lead to a considerable variation. Our study reported a 33% loosening rate according to mechanical measurement of extraction torque during instrumentation removal surgery. Meanwhile, we compared our extraction torque data with X-ray and CT scan findings, the result showed that the specificity of both imaging approaches were excellent, and the peri-screw osteolysis present both in X-Ray and CT scan could indicate low extraction torque of screw anchor, but their sensitivities were less than satisfactory (24% and 22% respectively), regarding detection of screw loosening. Furthermore, to our knowledge, for the first time we used extraction torque during screw removal surgery to analyze risk factors for screw loosening, our findings demonstrated that screws in non-fusion spine and fractured vertebrae had significantly lower extraction torque, while BMD and age showed low, but significant linear correlation with extraction torque.

A number of factors have been reported to be related to screw loosening. Excessive strain between the screw and bone interface is considered to be the primary cause for screw loosening [12, 22], which could be deteriorated when fusion is failure or the anterior support is inadequate. Meanwhile, stress shielding can lead to a decrease of stress transferred through the bone tissue, which can reduce bone mineral density and remodel the bone surrounding the screw. The presence of wear debris [23] was reported to induce osteolysis leading to screw loosening. The debris elicited an inflammatory cytokine-mediated particulate-induced response through increased expression of intracellular TNF-alpha, increased osteoclastic activity, and cellular apoptosis. Other factors that could cause bone loss or destruction, such as infection surrounding the implant, bone tumor, metabolic diseases, and microfracture due to excessive loading, are risk factors of pedicle screw loosening. Screw loosening may become a worsening problem due to the aging of population and the increasing number of osteopenic and osteoporotic patients. Wu et al. [17] reported higher occurrence of screw loosening in osteoporotic bone. In our study, we also found a significant linear correlation between BMD and extraction torque, indicating pedicle screws in aged patients or patients with lower BMD might be less stable due to lower extraction torque.

Our findings showed that X-ray had a sensitivity of 24% and a specificity of 98%, while CT scan had a sensitivity of 22% and a specificity of 95% regarding extraction torque as criterion of screw loosening. This result indicated that both radiological examinations were effective to confirm loosening screws; however, the low sensitivity implied a considerable number of loosening screws could be neglected by imaging study. Based on the torque data, the loosening rate was 33%, while both X-ray and CT scan only detected less than 30% of all loosening screws. Sanden [20] reported a 64% sensitivity of X-ray in 79 screws and a 35% rate of screw loosening, but their definition of a loosed screw was an extraction torque of 0.4 Nm or less as there were no screws with and extraction torque between 0.4 Nm and 0.75 Nm. We could not detect such a clear gap in our data. However, by using Twostep Cluster Analysis, we found the distribution of extraction torque data could be clustered into 3 clusters, with the cut-off value of 1.02 Nm and 2.22 Nm respectively. We also tested the average torque of newly implanted screws and found that 1.02 Nm was lower than the low limit of 95% confidence interval of newly implanted screws. Therefore, we set our cut-off torque at 1.02 Ncm for screw loosening. Although the torque values for screw loosening were different, the loosening rate of Sanden’s study based on extraction torque was similar to our findings, around 30–35%.

**Fig. 6:**
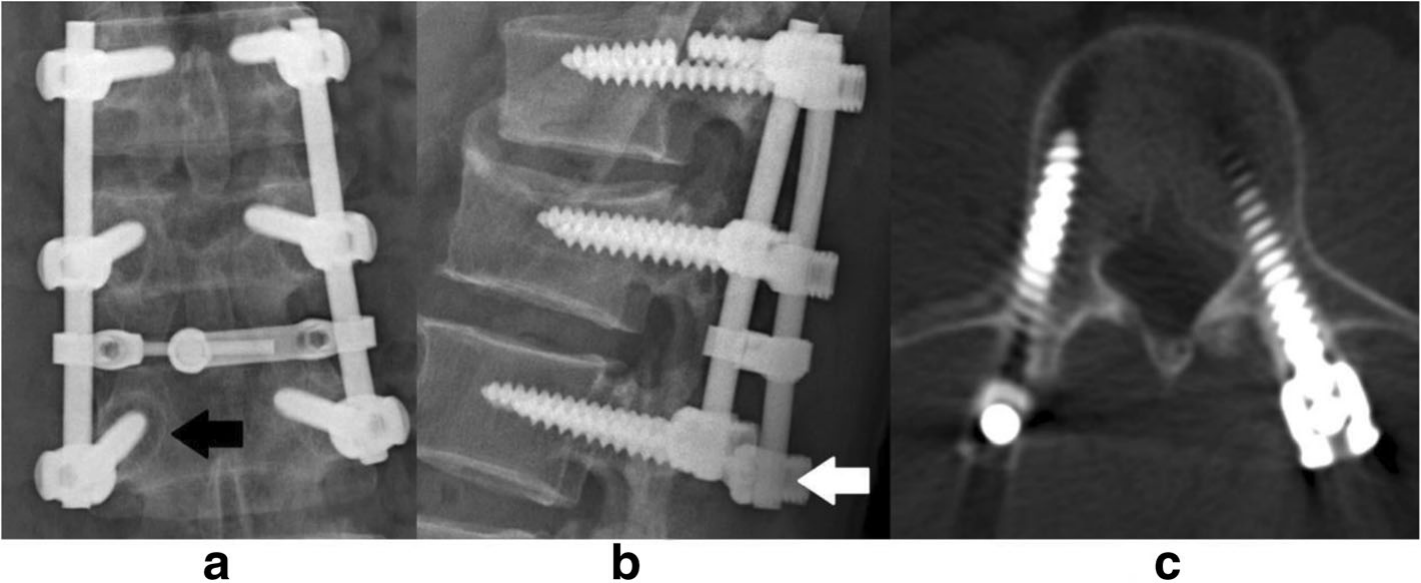
A typical case showing CT scan might be insensitive to detect screw loosening. **a** Anteroposterior X-ray showed radiolucent zone and double halo around the screw (black arrow) which indicate screw loosening. **b** Lateral X-ray showed that the same screw was pulled out (white arrow). **c** CT scan showed no gap around the same screw

Ohtori employed both CT scan and X-ray and the results showed that CT scan was more sensitive than X-ray [11]. In our study, there was no significant difference between X-ray and CT scan regarding both sensitivity (*p* = 0.863) and specificity (*p* = 1.00). It was observed in one case that CT scan failed to show a clear gap around screw, even though an obvious double halo was found on X-ray and the extraction torque was 0.06 Nm (Fig. 6). This could be resulted from the metal artifact that seriously interfered CT reconstruction and led to incorrect image surrounded metal instrumentation. Therefore, based on our result, CT might not be superior to X-ray in assessment of screw loosening, especially considering its higher cost and radiation exposure.

The risk factors of screw loosening we found based on extraction torque were fixation in fractured vertebrae and non-fusion spine. In the fractured vertebrae, the continuity of cortical and structure of trabecula were damaged, which might affect the stability of screw placed in it. Meanwhile, the strain imposed on pedicle screws might significantly increase when fusion had not been obtained or anterior column support was inadequate. We also found that pedicle screws in the lumbosacral junction (L4, L5, and S1) had significant higher extraction torque than those in other segments. A possible explanation could be that surgeries performed in lumbosacral spine were mainly for degenerative disc diseases, which often required spinal fusion, while in the thoracolumbar spine, where operations were more likely for vertebral fracture, fusion were not always necessary.

As expected, linear regression analysis showed that the stability of pedicle screw correlated positively with BMD and negatively with patients’ age. The relatively low related coefficients might be due to the existence of numerous confounding factors, and these results indicated that the failure risk of instrumentation increased with age and osteopenia/osteoporosis, which was widely credited but rarely proven in vivo with mechanical measurement.

Some reports showed that increasing length and diameter could increase the stability of pedicle screw [24, 25], which had not been observed from our results. The reasons that no significant different extraction torque was found among screws with different length and diameter might be due to the relatively small sample size and narrow range of length (30–55 mm) and diameter (4.0–6.5 mm). Further study with larger simple size is needed to confirm the effect of screw length and diameter on extraction torque in the human spine. Meanwhile, there was no significant difference in extraction torque regarding the screw design (polyaxial vs. monoaxial) and location (placed at the end segment vs. at the middle segment).

There are several limitations in our study. First, although this was a prospective and blinded designed study, the relatively small number of patients’ enrollment and the heterogeneity of screw size and position might render our findings susceptible to confounding factors. Hence, further studies with larger sample size and stratified data according to different factors will be needed to better understand the prevalent of pedicle screw loosening. Second, the lack of pedicle torque during primary surgeries made it unable to observe the longitudinal change of torque and the influence of inserting torque on screw loosening.

## Conclusion

In general, we found a 33% pedicle screw showed an extraction torque less than 1.02 Nm, which might be considered to be loosening according to our data distribution. Both X-ray and CT had high specificity to detect screw loosening, but their sensitivities could be overestimated. Surgeons need to be more cautious when assessing screw loosening based on radiological examination, since a considerable fraction of low extraction torque screws might have been underestimated. Pedicle screws of non-fusion surgery placed in fractured vertebrae had significantly lower extraction torque and, therefore, could be vulnerable to loosening. Pedicle screws in aged patients or patients with lower BMD might be less stable due to lower extraction torque.

## Abbreviations

BMD: Bone mineral density
CT: Computed Tomography
